# Implementation of World Health Organization behaviorally anchored rating scale and checklist utilization: promising results for LMICs

**DOI:** 10.3389/fmed.2023.1204213

**Published:** 2023-07-24

**Authors:** Syed Yousaf Khalid, Qazi Muhammad Sibghatullah, Muhammad Haroon Abdullah, Omer Farooq, Sandal Ashraf, Adeel Ahmed, Ashhar Arshad, Abdullah Nadeem, Hassan Mumtaz, Muhammad Saqib

**Affiliations:** ^1^Department of Surgery, Letterkenny University Hospital, Letterkenny, Ireland; ^2^Department of Surgery, District Headquarter Hospital, Rawalpindi, Pakistan; ^3^Department of Surgery, Fatima Memorial Hospital College of Medicine and Dentistry, Lahore, Pakistan; ^4^Department of Surgery, District Headquarter Hospital, Attock, Pakistan; ^5^Department of Surgery, Midland Regional Hospital Mullingar, Mullingar, Ireland; ^6^Department of Surgery, Gujranwala Medical College, Gujranwala, Pakistan; ^7^Department of Surgery, King Edward Medical University, Lahore, Pakistan; ^8^Department of Medicine, Dow University of Health Sciences, Karachi, Pakistan; ^9^Maroof International Hospital, Health Services Academy, Islamabad, Pakistan; ^10^Department of Emergency Medicine, Khyber Medical College, Peshawar, Pakistan

**Keywords:** WHOBARS, surgical safety, LMIC, quality improvement, humans

## Abstract

**Background:**

Operating teams can decrease the likelihood of patient risk by using the WHO Surgical Safety Checklist. To ascertain the impact of demographic factors on behaviorally anchored ratings and investigate operating room (OR) staff attitudes toward checklist administration, we set out to better understand how OR personnel use the checklist in a tertiary care hospital in Pakistan.

**Materials and methods:**

A monocentric sequential mixed-methods study employing a quantitative approach of using World Health Organization Behaviorally Anchored Rating Scale (WHOBARS) assessments of surgical cases by OR personnel and two independent observers, who were certified surgeons having extensive experience in the rating of the WHOBARS scale for more than 1 year, followed by a qualitative approach of staff interviews were carried out in a tertiary care setting. In June and July 2022, over the period of 8 weeks, an intervention (training delivery) was implemented and evaluated. The information, skills, and behavior adjustments required to apply the checklist were taught in the course using lectures, videos, small group breakouts, participant feedback, and simulations.

**Results:**

After the introduction of WHOBARS, 50.81% of respondents reported always using the checklist, with another 30.81% using it in part. Participants' years in practice, hospital size, or surgical volume did not predict checklist use. Checklist use was associated with always counting instruments (51.08%), patient identity (67.83%), difficult intubation risk (39.72%), the risk of blood loss (51.08%), prophylactic administration of an antibiotic (52.43%), and the use of pulse oximeter (46.75%). Interviewees felt that the checklist could promote teamwork and a safe culture, particularly enabling speaking up. Senior staff were of key importance in setting the appropriate tone.

**Conclusion:**

The use of a multi-disciplinary course for checklist implementation resulted in 50.81% of participants always using the checklist and an increase in counting surgical instruments. Successful checklist implementation was not predicted by the participant's length of medical service, hospital size, or surgical volume. If reproducible in other countries, widespread implementation in LMICs becomes a realistic possibility.

## Introduction

The occurrence of adverse events in a hospital context is currently estimated to be 10% globally (1), according to conclusive data. An estimated 234 million procedures are carried out worldwide per calendar year, according to reports. Of these, one million die, and there are thought to be seven million complications (2). Despite the possibility that inadequate hospital facilities and employees contributed to the increased mortality (3), up to 30% of contacts in the operating room (OR) suffer from communication breakdowns, which happen every 7–8 min on average (4). As a result, surgical care and the complications that come along with it carry a significant disease burden that requires the attention of the public health community on a global scale.

The WHO launched the Surgical Safety Checklist in 2008 as part of its second Global Patient Safety Challenge, Safe Surgery Saves Lives (5). The National Patient Safety Agency (NPSA) required the National Health Service (NHS) in England and Wales to use the checklist in January 2009 (6). The checklist's goal was to aid operating room (OR) teams in recalling crucial information that might be forgotten during an operation. Additionally, it was a tool for promoting communication and teamwork between OR staff members (5).

The World Health Organization Behaviorally Anchored Rating Scale (WHOBARS), a novel tool that evaluates behaviors related to the delivery of the checklist, was created to assess the level of engagement during the process (7). While using the checklist, health workers can be observed using the WHOBARS to evaluate their behavior. The effective participation of the entire OR team during the delivery of the WHO Surgical Safety Checklist (SSC) is necessary to realize its full potential to decrease perioperative damage. The overall structure of the checklist is left unchanged, but hospitals are invited to modify it to suit their needs. Studies proving the effectiveness of these modified checklists have persisted in demonstrating that using the SSC or a comparable checklist is generally advantageous (8).

One of the main justifications for checklists is that they may be used in varied contexts and significantly contribute to a reduction in morbidity and mortality related to surgery. The highest improvements are seen in low-income and middle-income countries (LMICs), where this checklist can cut mortality and morbidity after surgery by up to 50% (9). However, using the checklist incorrectly can have the opposite consequences (10). Although everyone demonstrates an understanding of the importance of this checklist, not everyone follows or uses it as intended (11). As a result, it is important to evaluate whether building an implementation strategy can help the checklist function sustainably.

This study was conducted in various healthcare facilities across Pakistan to assess the current utilization and effectiveness of the WHO surgical safety checklist. While the checklist is recommended in these settings, its implementation and adherence may vary. The study aimed to identify gaps, challenges, and potential areas for improvement in surgical safety practices by evaluating the utilization and effectiveness of the checklist. The findings will contribute to strategies and interventions aimed at enhancing the implementation of the WHO checklist in Pakistan, thereby improving patient safety outcomes. The rationale for this study was rooted in the recognition of the critical role that the checklist plays in improving patient safety during surgical procedures. Understanding the current situation in Pakistan and identifying barriers to checklist implementation provide valuable insights for developing targeted interventions and initiatives to enhance surgical safety practices and reduce preventable errors in the country. Ultimately, the goal was to promote the widespread adoption of the WHO checklist and foster a culture of patient safety in Pakistani healthcare settings.

## Methodology

This sequential exploratory mixed methods study with a sequential exploratory design where quantitative data were initially collected followed by qualitative data collection in the form of in-depth interviews was conducted over the course of 8 weeks starting from June to the last day of July 2022. The researchers obtained qualitative data from interviews and quantitative data using results from the WHOBARS rating as follows:

OR staff and two independent observers who were certified surgeons having extensive experience in rating the WHOBARS scale for more than 1 year used WHOBARS for the quantitative part of the study to rate the quality of checklist administration during surgical cases, answering questions 1 to 3. Descriptive statistics were used to analyze WHOBARS scores for secondary outcomes, and multivariate linear regression was used to identify which factors were significantly associated with checklist utilization. The use of checklists as measured by questionnaire was the primary outcome of interest in the quantitative statistical analysis. Covariates were included in the analysis for more accurate results. Multivariate linear regression was used for ranking questionnaire answers. The significance level for the analyzed data was set at 0.05, and the analysis was done using IBM Corp., released in 2013, IBM SPSS Statistics for Windows, version 22.0. Armonk, NY: IBM Corp.

Researchers conducted in-depth interviews lasting at least 1 h with more than 5 representative respondents from the OR staff who voluntarily presented themselves for the interview and who had completed the WHOBARS self-rating exercise for the qualitative part of the study to learn more about their thoughts and feelings regarding the checklist (research question 4) and its use in the OR. Interviews were conducted in Urdu and then translated into english and analyzed and categorized into overarching themes based on the six essential safety procedures for evaluating the effects of interventions. Both researchers were involved in the process of categorization and analysis.

Our study is fully compliant with the COREQ guidelines (12). A complete COREQ checklist has been provided as a Supplementary file. UIN research registry 8486 (13) identifies our study in Research Registry. Our research adheres to the principles outlined in the Helsinki Declaration.

All operating room staff was provided with pre-study presentations and information sheets, and their written consent was collected. Three operating rooms (ORs) were used, two for adults and one for children. To get a verbal agreement from patients (and/or their legal guardians), we provided them with information about the planned observations. If they did not want research staff to be present during their (or their child's) surgery, they were given the choice to opt out of the study.

### Public and patient participation

No members of the public or study participants were involved in any way. The research was performed in a tertiary care facility in the year 2022. Prompts to the checklist's three phases were posted on the walls of all operating rooms (ORs), as recommended by the Health Quality and Safety Commission's Safe Surgery initiative. Each step of the checklist was administered jointly. The anesthesiologist was in charge of sign-in, the surgeon of time-out, and the nurse of sign-out. Every person working in the operating room had never heard of WHOBARS and had no idea how to use it. The WHOBARS was introduced and used by the independent observers in the same manner as explained above. An academic pharmacist and a medical education researcher make up the two independent observers (trained in psychology). Neither of the outside evaluators had any experience in the operating room beforehand.

### Case selection

Based on the purposive sampling techniques of related studies (7, 14), we decided to investigate 20 full surgical cases. Adults and children alike who required emergency or elective surgery under general anesthesia during regular business hours were qualified. Once the daily list was up, the team of observers who had agreed to work in the operating room began their shift. Patients were assigned to any available OR based on the availability of medical professionals. To ensure that a wide variety of OR staff was represented, we limited daily observations to a single case from each OR. We did not include studies where either the staff or the patient refused to give their consent.

### Expertise development for raters

A total of 12 training videos were used in our prior studies and had already been rated by a group of trained raters before the ratings were done by the two independent observers. Across all 12 training clips, the two independent observers from this study and the trained raters from the original validation trial had an intraclass correlation coefficient of 0.84.

### WHOBARS ratings (quantitative)

In each phase of the checklist, two trained observers independently rated the five WHOBARS domains based on their observations of the entire case. Patients had to check in before anesthesia was administered, wait for a designated amount of time to pass before undergoing surgery, and then sign out before being allowed to leave the operating room. Following sign-out, the OR staff evaluated their team's performance using the WHOBARS instrument as well. We queried the OR staff for information such as gender, age, clinical specialty, and number of years of OR experience. The WHOBARS rating scale was employed by both the OR staff and outside observers. Rating guidelines were provided in great detail to the raters.

### Interviews (qualitative)

We then invited the OR staff in these situations to a semi-structured interview. Purposive sampling was employed here. There was a checkbox on the OR rater consent form that participants had the choice to indicate their willingness to be interviewed. A researcher with no prior connection to the participants contacted those who checked the box through email 2–6 days after they finished the OR ratings. We kept interviewing until we had enough information (when very few new ideas, opinions, or concepts were emerging from the interviews) and had a diverse sample of clinical roles and experience levels. To guarantee uniformity in the interview approach and ensure comparable interview results, all interviews were performed by the same researcher either in-person or over the phone (based on participant availability and preferences). During each interview, the researcher made careful notes of the interaction. The quotations may represent a selection, accurately reproduced, rather than the entire dataset.

### Data analysis

The primary result was examined only using descriptive statistics. Descriptive statistics were used to analyze WHOBARS scores for secondary outcomes, and multivariate linear regression was used to identify which factors were significantly associated with checklist utilization. The use of checklists as measured by the questionnaire was the primary outcome of interest in our study. The size of the hospital, the number of surgeries performed, the gender of the participants, their WHOBARS scores, how much they learned about patient safety, how satisfied they were with their jobs, and how much stress they felt they were under as a result of the training, all served as covariates.

We opted to use a hierarchical model instead. Likert scale answers were ranked from 0 to 4 for use in multivariate linear regression, with blanks being filled up with the mean score for that hospital. The significance level for the analyzed data was set at 0.05, and the analysis was done using SPSS version 22.

We translated all open-ended survey replies and all focus group information into English. Data were then analyzed using thematic analysis. Culture shift was analyzed using an inductive theme approach. Both researchers were involved in the process of identifying and emphasizing key issues, which were then categorized into overarching themes. We employed a deductive theme analysis, based on the six essential safety procedures for evaluating the effects of interventions.

## Results

### Quantitative results

In a 350-bed tertiary care hospital, we observed 160 cases. The final dataset included information from 370 people in 160 distinct cases. The sample includes 25 (6.75%) surgical assistants, 13 (3.54%) other health aid observers, 100 (18.37%) nurses, 68 (18.37%) anesthetists, and 164 (44.3%) surgeons. In Table 1, the participant demographics are displayed. No participant had received checklist training before the intervention. They all lacked enough pulse oximeters; therefore, the routine use of pulse oximetry could not be monitored. There was no regular counting of sponges, equipment, or needles. Additionally, the official procedure for debating the risk of challenging intubation, anticipated blood loss, or confirmation of antibiotic therapy was not followed. Respondents claimed that the patient's identification had been verified before the procedure.

**Table 1:** Participant demographics.

**Gender**	**Age**	**Clinical specialty**	**Number of years of OR experience**	**Hospital size**	**350 bedded**
Female	45	Pediatrics	10	Surgical volume	160
Male	52	Internal medicine	15	Number of participants	370
Female	47	Gynecology	12	Surgeons	164 (44.32%)
Female	44	Psychiatry	14	Anesthetists	68 (18.37%)
Male	50	Cardiology	18	Nurses	100 (27.02%)
Male	46	Ophthalmology	16	Surgical assistants	25 (6.75%)
Male	45	Orthopedic surgery	11	Other health aids	13 (3.54%)

Mean hospital WHOBARS scores and the method of evaluation are shown in Table 2.

**Table 2:** Mean hospital WHOBARS scores and the method of evaluation.

**Hospitals**	**Number of respondents per hospital**	**The method of evaluation**	**WHOBARS sign-in**	**WHOBARS time-out**	**WHOBARS sign-out**	**Overall WHOBARS score**
A	7	Real-time case in OR	7.2	6.8	7.0	7.0
B	8	Simulation	5.6	5.8	6.5	6.2
C	4	Total median scores (range) (IQR)	6.2 (1.6–7) (6–7)	5.8 (1.4–7) (5.8–7)	6.2 (1.6–7) (2.5–7)	5.6 (1.6–7) (4.9–7)

### Qualitative results

#### Anesthetist

Anesthetists used the checklist before the surgeon performed the surgical incision to fulfill their role. In collaboration with surgeons, they made sure to confirm the patient and type of surgery before each operation so that wrong-person or wrong-site surgery did not occur. It was not uncommon to assess risk for difficult intubation partly because of time constraints of the busy OR and partly because of a lack of skill and urgency of the severe disease requiring them to act rapidly and this important risk assessment was missed. This led to complications which then contributed to mortality. When everyone used the checklist, anesthetists said they felt much more like a responsible member of the team and that improved their morale. An anesthetist reported, “The checklist helps me be on track ensuring that I do not miss a preventable life-threatening situation”. The checklist promoted teamwork and aided the way forward for anesthetists. Anesthetists were thankful to their team members for giving them suggestions that saved lives. Anesthetists believed that vital information was overlooked as all eyes were not on the patient's ongoing surgical situation at any crucial moment in time during the surgery in OR, especially the sign-in and sign-out times.

#### Nurse

Although nurses are ordered to make sure every patient coming in for surgery has a pulse oximeter on them in the OR, they unfortunately lacked the logistics and enough oximeters which was the principal reason why not every patient was provided a pulse oximeter. Surgeons used swabs and instruments and nurses were given the job of correct counting to make sure that what goes in comes out and everything is accounted for but due to time constraints and fear of repercussions, especially from senior surgeons who have worked for a longer time in the OR, the nurses kept silent. According to an interview with a nurse, some members of the OR staff were downright sarcastic about it, which made genuine conversation about the checklist and patient safety more difficult than it already was at that moment in time. Nurses completed the sign-out when the surgeon left the OR. Surgeons only cared about the sign-in and they hastened the remaining procedure not giving nurses enough time to do everything appropriately. A nurse reported, “Surgeons would make jokes when I want to meticulously do each step of the checklist”. It used to really annoy nurses that some checklist users would just blindly tick that everything had all been done when it had not been done. The checklist enabled nurses to speak up for the patient and act in their best interests which was not possible before the implementation of the checklist.

### Anesthetic assistants, surgical assistants, and other health aids

Anesthetic assistants, surgical assistants, and other health aids remarked that nurses often had to remind the surgeons to complete components of the checklist before, but as time passed, everyone understood what to do and they all worked as a team. The checklist made it easy for anesthetic assistants, surgical assistants, and other health aids to raise concerns about patient safety and communication at different points for better patient care and healthcare delivery in general.

### Surgeons

Surgeons thought that it was only the job of the nurse to ensure every patient had a pulse oximeter in place in the OR. Surgeons posited that they generally advised antibiotic prophylaxis for every patient in the OR to prevent bacterial infections. One of the main reasons surgeons used antibiotics only half the time was because they thought administering antibiotics to every patient in the OR was not without its own downsides. They thought that blind antibiotic use in every patient coming in for surgery could lead to unwanted antibiotic resistance which could lead to the proliferation of superbugs that are more difficult to treat than the usual antibiotic-sensitive pathogens. Surgeons demonstrated an understanding that risk evaluation for blood loss had to be done for every patient, regardless of their status but that restricted their already constrained time allotted per surgery. Surgeons reported that they advised every serious patient ready supply of blood in case there arose a need for a blood transfusion during surgery. This protocol, however, was not followed for every patient due to the lack of resources. Every patient had different clinical status and not every patient required massive blood transfusions during surgery; hence, the risk of blood loss was evaluated only for serious patients. The most followed question of the checklist by surgeons was the identification of the patient and type of surgery with the anesthetist on board before a surgical incision was made. They stated that this was followed the most as they thought it was less time-consuming as well as the most yielding in terms of preventing never-events. Surgeons stated that certain individuals from the OR team appeared to be genuinely curious about the details and it seemed that some members of the OR staff were satisfied to simply go through the motions to get it over with as soon as possible, despite the fact that following such an approach completely undermined the purpose of the checklist exercise. For sign-outs, there was occasionally a sense of urgency as surgeons strived to finish the paperwork before the patient left the room. Obviously, there was a chance that something of importance could be overlooked if things were rushed, but it was assumed that such an event would not happen as much to cause an event of major clinical concern. A surgeon reported, “I am sure no life-threatening issue can arise if I just do the major things right”.

### Comparison between qualitative and quantitative results

Upon comparison of the quantitative results after the introduction of the WHOBARS rating and qualitative results from the interview, it is evident that the interviews provide insight into the data obtained using the quantitative method. We attempted to explain the quantitative data with the help of qualitative data under themes consistent with the questions of the checklist.

After the introduction of WHOBARS, 50.81% of respondents indicated that they always used the checklist in full (Table 3). With respect to performing the individual six basic safety processes, identification of the patient and type of surgery verified with surgeons on board before each operation (67.83%) was the most common to be done all the time. Evaluating the risk of difficult intubation before administration of anesthesia was the least commonly reported to be done all the time (39.72%). The frequency of self-reported use of the checklist and the six basic safety processes are shown in Table 3.

**Table 3:** Frequency of self-reported use of the checklist and the six basic safety processes.

**Question numbers**		**Always in full**	**Always in part**	**Sometimes**	**Occasionally**	**Never**	**No response**
1	Are you using the checklist in the operating room?	188 (50.81%)	114 (30.81%)	50 (13.51%)	7 (1.89%)	6 (1.62%)	5 (1.35%)
		**Always**	**Most of the time**	**Sometimes**	**Occasionally**	**Never**	**No response**
2	Identification of the patient and type of surgery verified with surgeons on board before each operation?	251 (67.83%)	74 (20%)	20 (5.40%)	11 (2.97%)	9 (2.43%)	5 (1.35%)
3	Risk of difficult intubation for the patient evaluated before giving anesthesia?	147 (39.72%)	54 (14.59%)	69 (18.64%)	49 (13.24%)	37 (10%)	14 (3.81%)
4	Risk of large blood loss evaluated before starting surgery?	189 (51.08%)	72 (19.45%)	49 (13.24%)	24 (6.48%)	17 (4.59%)	19 (5.16%)
5	Antibiotic prophylaxis was given before starting surgery?	194 (52.43%)	97 (26.21%)	38 (10.27%)	14 (3.78%)	17 (4.59%)	10 (2.7%)
6	Needles/swabs/ instruments counted before and after surgery?	189 (51.08%)	62 (16.75%)	74 (20%)	25 (6.75%)	19 (5.13%)	11 (2.97%)
7	Pulse oximeter being used in the operating theater?	173 (46.75%)	93 (25.13%)	26 (7.02%)	38 (10.27%)	28 (7.56%)	12 (3.24%)

A total of seven questions were asked and the responses were divided into six categories. The numbers without the brackets denote the sample size of respondents in the respective column and the numbers in brackets denote the percentage of those respondents among the total sample size.

### Using the checklist in the operating room

Results from the quantitative data show that 50.81% of respondents followed the checklist “always in full” and another 30.81% followed “always in part” after the introduction. In the interviews, respondents including nurses, surgeons, anesthetists, and anesthetic assistants mentioned that it played a positive role in their practice. Nurses and surgeons posited the fact that the use of a checklist helped OR staff recenters with a common goal of preventing adverse events and ensuring proper care of the patient. All members of OR who responded mentioned in one or another way that the use of a checklist helped them raise concerns, if any, that arose during the operation. Anesthetic assistants who responded also added that the use of checklists on a wider scale meant that they did not have to remind surgeons about checklist use since they already knew and implemented it in the OR.

### Identification of the patient and type of surgery

Results from the quantitative data show that 67.83% of respondents “always” followed the checklist about patient identity and type of surgery and another 20% followed it “most of the time” after introduction. In the interviews, surgeons and anesthetists mentioned that they did this as it was the easiest in terms of time consumption and the most yielding in terms of preventing never-events like the wrong-patient and wrong-site surgeries. Anesthetists echoed somewhat similar reasons and went on to mention that they did this as part of the sign-in process. When surgeons also did it as part of the sign-out process, this added extra safety and better prevented never-events.

### Evaluation of difficult intubation risk for the patient evaluated before giving anesthesia

Results from the quantitative data show that 39.72% of respondents “always” followed the checklist after the introduction and another 15.42% followed the checklist “most of the time” about the evaluation of difficult intubation risk for patients before giving anesthesia. In the interviews, anesthetists mentioned the reasons why this was the case and the main reason mentioned for using almost only half of the time was time constraints. The other reasons mentioned were lack of skill and the urgency with which the patient presented to the OR. A severely diseased patient requiring immediate surgery did not provide enough time to assess intubation risks in detail as the goal is to do surgery as quickly as possible.

### Evaluation of the risk of large blood loss before starting surgery

Results from the quantitative data show that 51.08% of respondents “always” followed the checklist about the risk of large blood loss during surgery and another 19.45% followed it “most of the time” after introduction. In the interviews, surgeons demonstrated the knowledge of the significance of evaluation for the risk of large blood loss for every patient. Reasons that acted as a hindrance to 100% adoption of this practice include, but are not limited to, lack of time, resources, type of surgery, clinical status, and the general thought that not every patient needs massive blood transfusions.

### Antibiotic prophylaxis before surgery

Results from the quantitative data show that 52.43% of respondents “always” followed the checklist after the introduction regarding the administration of prophylactic antibiotics before surgery and another 26.21% of respondents followed it “most of the time”. In the interviews, surgeons posited that they generally advise antibiotic prophylaxis for patients in the OR to prevent bacterial infections but the fear of unwanted antibiotic resistance and the proliferation of superbugs prevented them from adopting it 100% of the time.

### OR material count before and after surgery

Results from the quantitative data show that 51.08% of respondents “always” followed the checklist after the introduction regarding instruments, swabs, and needles count and another 16.75% of respondents followed it “most of the time”. In the interviews, nurses reported that their job of correct counting of instruments before and after surgery could not be followed all the time due to time constraints and fear of repercussions, especially from senior surgeons who have worked for a longer time in the OR. Surgeons on the other hand stated that they tried and made sure not to leave instruments inside but the complex OR environment and lack of communication led to adverse events such as leaving surgical instrumentation inside a patient's body after surgery is over.

### Use of pulse oximeter in the OR

Results from the quantitative data show that 46.75% of respondents “always” followed the checklist after the introduction about the use of pulse oximeter in the OR and another 25.13% of respondents followed it “most of the time”. In the interviews, respondents stated that they try their best to ensure every patient has a pulse oximeter in place while they are in the OR but the principal reason that this is not brought to fruition is lack of resources.

## Discussion

In this article, we report a longitudinal investigation into the steady use of the Surgical Safety Checklist in Pakistan. According to our knowledge, this is one of the first in-depth analyses of checklist usage and surgical safety procedures conducted in Pakistan. Following the introduction of WHOBARS, the checklist was used to a modest extent, with 50.81% of participants completing it entirely. Hellar et al. (15) noted a higher SSC use rate of 68.8–99.4% (15) in Tanzania. Another study reveals that the checklist is used 65% of the time on average (16). The WHO checklist promotes adherence to fundamental safety procedures and seeks to increase operating room safety by fostering better cooperation and communication. Traditional medical training places a strong emphasis on the need for error-free practice and employs strong peer pressure to ensure accuracy in both diagnosis and treatment (17). Failures in communication can result in avoidable patient damage on their own. They can also be the root cause of subsequent injuries. Medicine errors can result from inadequate communication among doctors, pharmacists, nurses, and patients regarding the name, dose, delivery method, and timing of medication administration (18). Adherence and teamwork were listed by Treadwell et al. (19) as determinants of successful SSC results (19). Greater situation awareness is also made possible by communicating task-related information (20).

Implementing checklists in LMICs is hampered by a hierarchical culture, a lack of resources, and a lack of understanding (21). The first step in eliminating retained surgical materials is accurate counting and documentation, although this is rarely done in many LMICs. Counting needles, swabs, and other tools, as well as the scheduling of antibiotic prophylaxis, came up frequently in our focus group discussions. Our research showed that 52.43% of individuals considered evaluation of significant blood loss before surgery began. Additionally, 51.08% of participants in our study counted needles, swabs, and equipment both before and after surgery. We discovered that 46.75% of participants regularly used pulse oximeters in operating rooms. Due to the significant percentage of “no responses,” it is assumed that these fundamental safety measures are implemented less frequently than 50.81% of the time. Therefore, there is still room for development, and future checklist courses ought to emphasize this communication-related aspect. The findings of this study, when compared to other studies, reveal both similarities and differences. Similar to previous research, the completion rate of the checklist in this study was around 50.81%, which aligns with the range of checklist usage rates reported in other studies. The importance of communication and teamwork in enhancing operating room safety was emphasized in both this study and previous research. Both highlighted the negative consequences of communication failures and the positive impact of adherence to teamwork principles. Additionally, the need for improvement in implementing fundamental safety measures was a common theme. Inadequate counting and documentation of surgical tools, evaluation of blood loss, and the consistent use of pulse oximeters were identified as areas where enhancements can be made. However, this study also highlighted the specific challenges faced in low- and middle-income countries (LMICs), such as hierarchical cultures, resource limitations, and lack of understanding. These challenges were not explicitly mentioned in other studies. The use of focus group discussions in this study provided qualitative insights that complemented the quantitative data from other studies. Overall, the findings from this study support the existing knowledge about the importance of checklist implementation and teamwork while identifying specific areas for improvement, particularly in communication-related aspects, to enhance operating room safety in the future.

The use of checklists was linked to a better understanding of patient safety, hospital size, surgical volume, WHOBARS, greater personal satisfaction, and lower workplace stress. We were able to comprehend the dynamics underlying the OR rater scores, thanks to in-depth interviews. The critical roles of senior physicians were the quality of checklist administration, allowing staff to speak up, and connecting checklist administration to patient outcomes. These were also some of the elements our qualitative research discovered as reasons explaining both positive and negative staff impressions. We think that if used by OR personnel, self-ratings of checklist administration using this tool that specifically describes these behaviors might help to improve them in the administration of the checklist. Participants emphasized the need for staff members to actively participate in the checklist as part of a genuine conversation to prevent it from degenerating into a pointless exercise.

The checklist, according to nurses and anesthetists, was important in forming the team and bringing everyone together. In a Finnish trial, survey questions were distributed to treating personnel before and after the WHO checklist was implemented. Their understanding of team member names and responsibilities as well as the patient's identity, history, medications, and allergies significantly improved (22). Nevertheless, they noted that senior consultants did not take the checklist seriously and did not engage in full participation. Our research revealed that nurses frequently felt hesitant to voice their concerns.

Communication errors may be significantly influenced by a reluctance to speak up (23). The degree to which a nurse speaks up has been shown by Kolbe et al. to be a predictor of technical team performance (24). Additionally, we discovered that surgeons readily voice their worries since they believed it was their obligation. We discovered that only 39.72% of participants thought about the possibility of challenging intubation before administering an anesthetic.

On inquiring the operating staff about potential obstacles to adopting the checklist, we learned that ignorance of the checklist, a lack of knowledge of how to use it, a lack of drive, and a sense of its insignificance were the main obstacles. This finding is in contrast to research conducted in England, which found that the most frequent obstacle to the checklist's execution was opposition from senior faculty (25) followed by a shortage of staff members with the necessary knowledge and training. The opposite conclusions were drawn by a second French investigation as well. Some components of the checklist appeared to be consistently covered by pre-existing processes in surgical settings, making their adoption ineffectual and staff members' perceptions of it as checklist duplication unfavorable. Other obstacles were a lack of team member communication and the notion that the checklist was pointless and laborious to complete (26).

To improve patient safety, the WHO created the Surgical Safety Checklist. However, there was not enough knowledge about the formal execution of the checklist in our operating rooms inside our organization. Therefore, the main goals of our quality improvement project were to evaluate the level of adherence to the WHO Surgical Safety Checklist components within our current practice and to increase knowledge of its application. The ultimate goal of this research was to advance patient safety standards by determining any potential improvement in compliance following our educational intervention.

Comparing the findings in our study to those of other studies, several similarities and differences can be observed. Both our study and other research emphasize the importance of checklists in improving patient safety and enhancing team collaboration. The use of checklists was associated with a better understanding of patient safety, increased personal satisfaction, and reduced workplace stress in our study. Similarly, other studies found that checklists improved the understanding of patient information and communication within the team. However, there are differences in the identified obstacles to checklist adoption. Our study highlighted issues such as ignorance, lack of knowledge, and perceived insignificance, while other studies mentioned opposition from senior faculty and a shortage of trained staff. Additionally, our study revealed hesitancy among nurses in voicing concerns, which aligns with other research indicating that reluctance to speak up can lead to communication errors. These findings highlight the importance of addressing barriers to checklist implementation and promoting a culture of open communication within healthcare teams.

Additionally, our study has several drawbacks. A major drawback of our mixed method approach to this study is that qualitative information on a specific person's beliefs, experiences, and behaviors could not accurately reflect the general group effect predicted by a larger sample size in the quantitative portion of the study. Another drawback of our study is that interview partner selection was not random. It was voluntary and interviews were only conducted on those who voluntarily opted to give an interview. This is a potential source of selection bias in our study. Future studies should focus on random partner selection to eliminate this bias.

## Conclusion

Our study's findings indicate that after the introduction of WHOBARS, 50.81% of participants use the WHO surgical safety checklist “always in full” every time. The use of a multi-disciplinary course for checklist implementation resulted in 50.81% of participants always using the checklist. If reproducible in other countries, widespread implementation in LMICs becomes a realistic possibility. Early results from the training course indicate the potential for widespread checklist implementation and counting instruments in LMICs, but more study is required to assess long-term sustainability.

## Data availability statement

The raw data supporting the conclusions of this article will be made available by the authors, without undue reservation.

## Ethics statement

The studies involving human participants were reviewed and approved by the Ethical Review Board of LNUH dated, 1st June 2022 ref no ERC: DSWIRB/2022/03061. The patients/participants provided their written informed consent to participate in this study.

## Author contributions

All authors have equally contributed to the manuscript and have approved the final manuscript to be published.
